# Preventing graft re-JAK-tion: safer transplant conditioning enables murine islet allograft tolerance and diabetes reversal

**DOI:** 10.1172/JCI201105

**Published:** 2026-01-02

**Authors:** Stephen P. Persaud, John F. DiPersio

**Affiliations:** 1 Division of Laboratory and Genomic Medicine, Department of Pathology & Immunology, and; 2 Section of Cellular Therapy, Division of Oncology, Department of Medicine, Washington University School of Medicine, St. Louis, Missouri, USA.

## Abstract

Transplantation of allogeneic islets of Langerhans, which include the insulin-producing β cells of the endocrine pancreas, holds curative potential for type 1 diabetes (T1D). However, protecting the allograft from the host immune system has long been a challenge impeding wider use of this therapy. Inducing mixed hematopoietic chimerism via allogeneic hematopoietic stem cell transplantation (HSCT) can achieve long-lasting donor-specific immune tolerance, but the toxicities of conventional HSCT conditioning agents limit the use of this approach. In this issue of the *JCI*, Bhagchandani et al. have used the JAK1/2 inhibitor baricitinib to optimize a nonmyeloablative antibody-based HSCT conditioning regimen, achieving multilineage hematopoietic engraftment, which enabled curative islet allotransplantation in a mouse model of T1D.

Human islet pancreatic transplantation, first described in 1989 by Scharp, Lacy, and colleagues (1), has the potential to cure T1D and eliminate affected individuals’ lifelong need for insulin therapy. However, islet rejection remains a major hurdle to successful transplantation, necessitating long-term immunosuppression. Adding further complication, the drugs that suppress islet rejection can be toxic to β cells and increase risk of cancer and infection (2). The serious adverse effects of systemic immunosuppression regimens have motivated development of alternative strategies that promote durable immune tolerance in transplant recipients.

## Allogeneic HSCT for tolerance induction

Allogeneic HSCT (allo-HSCT) holds vast therapeutic potential for numerous diseases of the blood and immune system. Moreover, allo-HSCT before solid organ transplantation (SOT) can engraft the immune system of the donor into the recipient, resulting in stable mixed-donor chimerism that enables a lasting state of tolerance to the transplanted organ. However, the potential benefits of allo-HSCT must be weighed against the treatment-related toxicities caused by the chemotherapy and/or irradiation that are conventionally used as transplant conditioning agents. Indeed, the potential for severe adverse events resulting from HSCT conditioning may partially explain why allo-HSCT is typically reserved for the most life-threatening clinical indications, such as acute myeloid leukemia (AML) and other hematologic malignancies. However, in elderly or infirm patients for whom allo-HSCT may be otherwise indicated, the risk of adverse treatment-related events might preclude them from being candidates for this potentially lifesaving therapy altogether. Therefore, overcoming the barriers to safe and effective allo-HSCT imposed by conditioning-related toxicities would broaden the patient populations and clinical indications in which transplantation may safely be used, including the induction of donor-specific tolerance to prevent islet allograft rejection.

## Minimally toxic, CD117 antibody–based conditioning

There is considerable interest in developing targeted, minimally toxic conditioning agents for HSCT that avoid the systemic toxicities of chemotherapy and irradiation-based conditioning. We and others have evaluated antibody-drug conjugates (ADCs) targeting the tyrosine phosphatase CD45 (3, 4) and the tyrosine kinase CD117 (also called c-Kit) (5, 6) in preclinical models. These studies have found these agents to be generally well tolerated and efficacious for transplantation both within (syngeneic and autologous gene therapy) and across (allogeneic) immunological barriers, enabling amelioration of disease in various models of malignant (7, 8) and nonmalignant hematologic disease (3, 9). Moreover, regimens using naked antibodies, which avoid the potential toxicities of ADC payloads, have been described in which targeting CD117 alone or in combination with blockade of the CD47/SIRPα axis successfully permitted disease correction in mouse models of chronic granulomatous disease (10) and myelodysplastic syndrome (MDS) (11). Notably, the anti-CD117 antibody briquilimab has shown efficacy as a conditioning agent in clinical trials involving patients with Fanconi anemia (12), MDS/AML (13), and severe combined immunodeficiency (14).

## Exploring JAK inhibitors to fight graft rejection

Graft rejection by the host adaptive immune system, which is mediated primarily by T lymphocytes and NK cells, is a critical barrier to engraftment. This is a particularly important consideration for antibodies and ADCs that are unable to sufficiently suppress these cell populations on their own, since alloengraftment would then require further immunosuppression. Recently, we reported that balanced JAK1/2 inhibitors, which include drugs like ruxolitinib and baricitinib, permit stable multilineage engraftment when combined with either ADC- or naked antibody–based conditioning in mouse models (4, 10). A notable advantage of the use of JAK inhibitors in the setting of transplantation is their potential utility in preventing both host-versus-graft responses that cause rejection as well as the pathologic graft-versus-host responses that underlie graft-versus-host disease (GvHD), the major cause of post-HSCT morbidity and mortality. Interestingly, unlike conditioning with irradiation, we have not observed GvHD in mice conditioned with either naked antibodies or ADCs; this has held true when transplanting T cell–replete donor marrow or even upon infusion of allogeneic splenocytes containing large numbers of alloreactive T cells (4).

**Figure 1:**
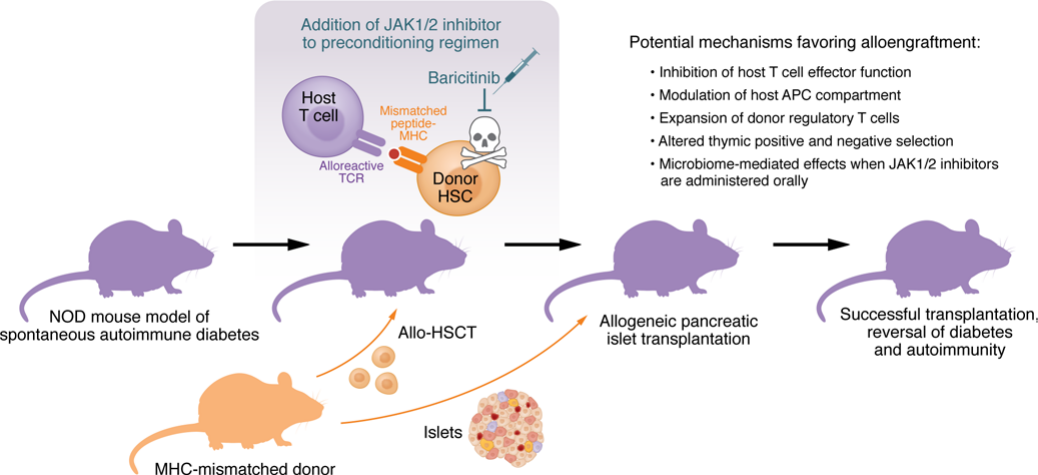
Exploring JAK1/2 inhibition as a single-agent immunosuppression regimen to enable allo-HSCT and subsequent islet transplantation. Bhagchandani et al. (16) showed that adding subcutaneous injections of the JAK1/2 inhibitor baricitinib to an allo-HSCT– and anti-CD117–based preconditioning regimen enabled successful pancreatic islet transplantation and diabetes reversal in the NOD mouse model of spontaneous autoimmune diabetes. In previous studies, JAK1/2 inhibition with oral ruxolitinib alone overcame T cell– and NK cell–mediated rejection to enable fully mismatched allo-HSCT when combined with CD45- and CD117-targeted ADCs (4). This suggests that JAK1/2 inhibition alone may enable TBI-free allo-HSCT in the NOD mouse model that then allows for curative islet transplantation and diabetes reversal. The mechanisms by which JAK1/2 inhibitors prevented T cell–mediated rejection remain incompletely defined. Notably, in prior work, a short peritransplant course of JAK1/2 inhibition enabled the HSC allograft to persist months after the drug was stopped, suggesting effective induction of peripheral and central tolerance that prevented residual host T cells or de novo–generated T cells, respectively, from depleting allogeneic HSCs or their progeny (4, 10).

We have explored the use of JAK1/2 inhibitors as immunosuppressants for SOT in skin and heart transplant models (15). However, except for skin graft studies, the use of JAK1/2 inhibitors and antibody-based conditioning had not been extensively investigated in the context of allo-HSCT for donor-specific tolerance induction. Herein, Bhagchandani et al. (16) have incorporated the JAK1/2 inhibitor baricitinib into a combination immunosuppression regimen for eliciting donor-specific allotolerance toward pancreatic islets of Langerhans in a mouse model of T1D (Figure 1).

## Combination regimen overcomes poor engraftment in T1D model

The study by Bhagchandani et al. (16) builds upon prior work from the same group. In 2022, Chang et al. (17) combined anti-CD117 with T cell–depleting antibodies and low-dose total-body irradiation (TBI) (300 cGy) to achieve fully mismatched HSCT (BALB/c mice to B6 mice) that allowed for stable engraftment of donor- or recipient-derived, but not third party–derived, islets. Alloengrafted islets restored normoglycemia to RIP-DTR mice, an inducible model of T1D in which mice are rendered insulin deficient via diphtheria toxin–mediated ablation of pancreatic β cells. In the present work, Bhagchandani et al. (16) aimed to address a key next step: to therapeutically intervene in spontaneous diabetes, which is faithfully modeled by the NOD mouse strain. However, when they adopted a similar strategy to Chang et al. (17) — performing allo-HSCT then islet transplantation after a nonmyeloablative anti-CD117–based regimen — it was initially unsuccessful in the NOD background due to poor HSC engraftment. Considering that this outcome could be attributable to the reported radioresistance of the T cell compartment in NOD mice (18), Bhagchandani et al. (16) overcame this barrier by adding daily subcutaneous injections of the JAK1/2 inhibitor baricitinib to their regimen during the peritransplant period. In so doing, they not only were able to achieve stable multilineage donor hematopoietic chimerism that enabled subsequent islet alloengraftment, but could reduce the TBI dose compared with that used in the study by Chang et al. (17). Importantly, although the conditioning regimen alone (without HSCT) seemed to delay the onset of overt diabetes, the authors were able to demonstrate via tetramer staining and adoptive transfer experiments that allo-HSCT, but not conditioning alone, was required to purge diabetogenic clones from the T cell repertoires of the islet transplant recipients.

## Unpacking JAK inhibition’s effects in tolerance induction

How JAK1/2 inhibition enables HSC engraftment in allo-HSCT remains incompletely understood. Prior experiments done in syngeneic HSCT showed no benefit of adding baricitinib or ruxolitinib, suggesting that the primary effect of JAK1/2 inhibition in allo-HSCT is to provide immunosuppression (4, 10). Still, given that most cytokines signal via JAK1- and/or JAK2-dependent pathways, narrowing down the mechanisms that are relevant to enabling alloengraftment remains challenging. Another open question is whether individual JAK inhibitor compounds differ from one another in their efficacy as HSCT conditioning immunosuppressants. The choice of inhibitor is clinically relevant, as HSCT physicians have considerably more experience with ruxolitinib (as therapy for GvHD) than with baricitinib; of note, baricitinib has a black box warning for serious thrombotic events, which may disfavor its prolonged use during HSCT (19). Finally, although the present results were obtained by adding daily JAK1/2 inhibitor injections to low-dose TBI and T cell depletion for allo-HSCT, it is intriguing to consider whether the JAK1/2 inhibitor alone might be sufficient for this effect. Indeed, we have found that stable, fully mismatched mouse allo-HSCT after ADC and naked antibody conditioning can be achieved without any irradiation or lymphodepletion by using an oral formulation of ruxolitinib. Importantly, use of a continuous oral dosing format was essential for achieving robust engraftment across immunological barriers, as neither daily JAK1/2 inhibitor injections or even drug delivery via osmotic pumps were sufficient (4). Application of an orally formulated JAK inhibitor in lieu of daily drug injections may further improve upon the regimen reported by Bhagchandani et al. (16) by removing the requirement for T cell depletion and TBI altogether.

## Looking ahead to safe and effective islet transplantation

In summary, the study by Bhagchandani et al. (16) comes at an exciting time for cellular therapy and regenerative medicine for T1D. Although human islet transplantation was first described over 35 years ago (1), the challenges of obtaining enough islets to transplant and protecting their long-term function in vivo have long been major barriers to wider adoption of this modality as a definitive therapy. Recent advances in generating allogeneic stem cell–derived islets and genetically modifying them to avoid detection by the host immune system (20) suggest the feasibility of off-the-shelf islet therapeutics for T1D, spurred on by early clinical successes in achieving prolonged insulin independence in patients (21, 22). A potential advantage of using HSCT for tolerance induction is that resetting the immune system in this way may correct the underlying issue of autoimmunity, which would be of particular benefit in patients whose immunopathology extends beyond the islets to involve other endocrine glands, skin, the gastrointestinal tract, or other tissues. Future basic, translational, and clinical studies in this promising field will contribute to the ultimate goal of broadening the safe application of islet transplantation to improve patient access to this potentially life-changing therapy.

## Funding support

This work is the result of NIH funding, in whole or in part, and is subject to the NIH Public Access Policy. Through acceptance of this federal funding, the NIH has been given a right to make the work publicly available in PubMed Central.

- National Cancer Institute (NCI) Outstanding Investigator Award (R35CA210084 to JFD, including a research supplement to promote diversity to SPP).
- National Heart, Lung, and Blood Institute Career Development Award (K08HL168155 to SPP).
- NCI Specialized Program of Research Excellence (SPORE) grant in leukemia (P50CA171963 to JFD).
- SPORE Career Enhancement and Developmental Research Award (P50CA171063 to SPP).
- American Society for Transplantation and Cellular Therapy New Investigator Award (to SPP).
- American Society of Hematology Scholar Fellow-to-Faculty Award (to SPP).
- Gabrielle’s Angel Foundation for Cancer Research awards (to SPP).

## Version 1 01/02/2026

Electronic publication
